# Draft genome sequence of *Muricoccus roseus* DSM 14916^T^, isolated from a children’s care center in Finland

**DOI:** 10.1128/mra.00891-25

**Published:** 2025-11-26

**Authors:** Alejandra Valenzuela, Jair Cortez, Janelle Mande, Cody Fondse, Vera Thiel, Kurt LaButti, Nikos Kyrpides, Rekha Seshadri, Tricia A. Van Laar

**Affiliations:** 1 Department of Biological Sciences, California State University, Stanislaus, Turlock, California, USA; 2 Leibniz Institute DSMZ—German Collection of Microorganisms and Cell Cultures GmbH, Braunschweig, Germany; 3 DOE Joint Genome Institute, Lawrence Berkeley National Laboratory 1666, Berkeley, California, USA; Queens College Department of Biology, Queens, New York, USA

**Keywords:** *Muricoccus*, taxonomy, phylogeny, *Roseomonas*, *Pararoseomonas*

## Abstract

*Muricoccus roseus* DSM 14916^T^, a strain also reported as *Roseomonas rosea* and *Pararoseomonas rosea*, was isolated from a water-damaged childcare center in Finland. We report the whole-genome sequence and annotation, identifying genes associated with carotenoid biosynthesis. This sequence may be useful for studies on taxonomic clarification and environmental microbiology.

## ANNOUNCEMENT

*Muricoccus roseus* DSM 14916^T^, a pink-pigmented, Gram-negative bacterium, was isolated from a water-damaged wall of a childcare center in Finland (1) and classified as a novel genus and species based on 16S rRNA sequencing and physiological characteristics (2). Subsequent proposals placed this species in *Roseomonas* (3) and *Pararoseomonas* (4), but those reclassifications, published under the International Code of Nomenclature of Prokaryotes (5), are considered illegitimate by the List of Prokaryotic names with Standing in Nomenclature (6, 7). This inconsistency is highlighted by polyphyly of *Roseomonas* in Bergey’s Manual (8). The genome of *M. roseus* DSM 14916^T^ was sequenced as part of the Genomic Encyclopedia of Bacteria and Archaea project to broaden genomic resource diversity (9).

DSM 14916^T^ was cultured aerobically for 4 days in TSB at 28°C and 100 rpm at the DSMZ. Biomass was harvested by centrifugation, and DNA was extracted using the MasterPure DNA Purification Kit (Lucigen) per the manufacturer’s instructions. One hundred nanograms of DNA was sheared to 270 bp using Covaris LE220 and size-selected with SPRI beads (Beckman Coulter). Fragments underwent end-repair, A-tailing, and adapter ligation (IDT, Inc.) using KAPA-Illumina Library Creation Kit (KAPA Biosystems). The library was quantified using KAPA Library Quantification Kit (Roche) on Roche LightCycler. The library was then multiplexed with others and prepared for sequencing on the Illumina HiSeq sequencing platform utilizing a TruSeq Paired-end Cluster Kit, v3, and Illumina’s cBot instrument to generate a clustered flow cell. Sequencing was performed on an Illumina HiSeq 2000 using HiSeq TruSeq SBS sequencing kits, v3, with a 2 × 150 indexed run recipe. DUK, part of BBtools, was run with default parameters for quality, adapter, and contaminant trimming (10). Reads were assembled using Velvet (v.1.2.07; velveth: 63 –shortPaired and velvetg: –very clean yes –exportFiltered yes –min contig lgth 500 –scaffolding no –cov cutoff 10) (11). Simulated 1–3 kb paired-end reads were created with wgsim (–e 0 –1 100 –2 100 –r 0 –R 0 –X 0) (12). Simulated and Illumina reads were assembled using Allpaths-LG (v.r46652; PrepareAllpathsInputs: PHRED 64=0 PLOIDY=1 FRAG COVERAGE=125 JUMP COVERAGE=25 LONG JUMP COV=50, RunAllpathsLG: THREADS=8 RUN=std shredpairs TARGETS=standard VAPI WARN ONLY=True OVERWRITE=True). The genome was 96.89% complete with 0.16% contamination via CheckM2 v.1.0.2 (13). Genome annotation used the JGI Microbial Genome Annotation Pipeline v.4 (14) with the standard DOE-JGI protocol. Results were integrated into the Integrated Microbial Genomes system for comparative analysis (15).

**Fig 1:**
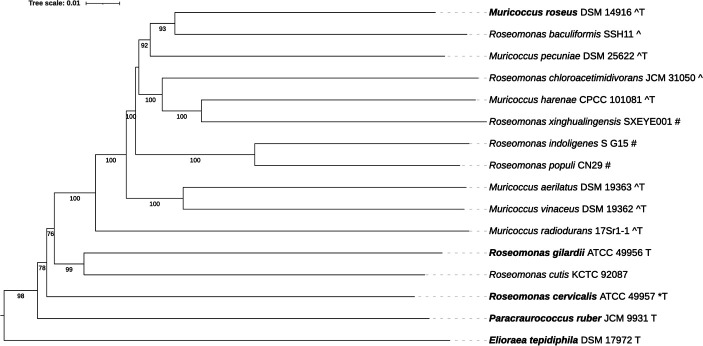
Tree inferred with FastME 2.1.6.1 (19) from Genome BLAST Distance Phylogeny (GBDP) distances calculated from genome sequences. The branch lengths are scaled using GBDP distance formula *d_5_*. Numbers are GBDP pseudo-bootstrap support values >60% from 100 replications, with an average branch support of 92.5%. The tree was rooted using *Elioraea tepidiphila* as an outgroup (20). Bold, type species for genus; T, type strain for species; *, recommended reclassification to *Pseudoroseomonas* (4); ^, recommended reclassification to *Pararoseomonas* (4); #, likely *Pararoseomonas* but sequenced after Rai et al. (4).

The final genome assembly was 5,342,582 bp with a G+C content of 70.76% and 5,027 predicted protein-coding genes (Table 1). A phylogenetic tree was generated using the Type Strain Genome Server (16) and visualized using the Interactive Tree of Life v.6 (17) (Fig. 1). The closest relative of *M. roseus* was *R. baculiformis* (44.5% dDDH), with 17.3% similarity to *R. gilardii*, supporting its placement outside the genus *Roseomonas*. Given this isolate’s pigmentation, we used antiSMASH (v.8.0) (18) which detected carotenoid biosynthesis genes, including *crtB* (*BUB97_RS25410*), *crtI* (*BUB97_RS02570*), and *crtX* (*BUB97_RS02555*). Additional clusters included a hydrogen cyanide operon (*hcnABC: BUB97_RS10025*, *BUB97_RS10030*, *BUB97_RS10035*) and a non-ribosomal peptide synthetase (*BUB97_RS12185*), suggesting potential for antimicrobial and bioactive compound production.

**TABLE 1:** Genomic features of *Muricoccus roseus* DSM 14916^T^

Feature	Finding
Genome size (bp)	5,342,582
Number of scaffolds	81
Scaffold N50 (bp)	151,080
Scaffold L50 (kb)	12
GC content (%)	70.76
Genome coverage	210.8×
No. of protein coding genes	5,027
tRNA genes	47
rRNA genes (5S, 16S, and 23S)	7 (1, 1, 5)

## Data Availability

This Whole Genome project has been deposited in GenBank under accession no. FQFZ00000000. The version described in this paper is the first version, FQZF01000000. The raw reads have been deposited in the NCBI Sequence Read Archive (SRA) under the accession number SRX2066078. Additional data can be explored or downloaded from the JGI Integrated Microbial Genomes with Microbiomes (IMG/M) portal using the taxon ID 2585427600.
